# A New Noncanonical Anionic Peptide That Translocates a Cellular Blood–Brain Barrier Model

**DOI:** 10.3390/molecules22101753

**Published:** 2017-10-18

**Authors:** Sara Neves-Coelho, Rute P. Eleutério, Francisco J. Enguita, Vera Neves, Miguel A. R. B. Castanho

**Affiliations:** Instituto de Medicina Molecular, Faculdade de Medicina, Universidade de Lisboa, Av. Prof. Egas Moniz, Lisboa 1649-028, Portugal; sncoelho@medicina.ulisboa.pt (S.N.-C.); ruteleuterio@gmail.com (R.P.E.); fenguita@medicina.ulisboa.pt (F.J.E.); macastanho@medicina.ulisboa.pt (M.A.R.B.C.)

**Keywords:** cell-penetrating peptide, blood–brain barrier, anionic peptide, drug-delivery systems

## Abstract

The capacity to transport therapeutic molecules across the blood–brain barrier (BBB) represents a breakthrough in the development of tools for the treatment of many central nervous system (CNS)-associated diseases. The BBB, while being protective against infectious agents, hinders the brain uptake of many drugs. Hence, finding safe shuttles able to overcome the BBB is of utmost importance. Herein, we identify a new BBB-translocating peptide with unique properties. For years it was thought that cationic sequences were mandatory for a cell-penetrating peptide (CPP) to achieve cellular internalization. Despite being anionic at physiological pH, PepNeg (sequence (SGTQEEY) is an efficient BBB translocator that is able to carry a large cargo (27 kDa), while maintaining BBB integrity. In addition, PepNeg is able to use two distinct methods of translocation, energy-dependent and -independent, suggesting that direct penetration might occur when low concentrations of peptide are presented to cells. The discovery of this new anionic *trans*-BBB peptide allows the development of new delivery systems to the CNS and contributes to the need to rethink the role of electrostatic attraction in BBB-translocation.

## 1. Introduction

Neurological disorders are a significant public health concern, being related to 12% of worldwide population deaths. Additionally, with lifetime expectancy growth and global population aging, there is an increase in the prevalence of neurological disorders in both developed and developing countries [1]. Besides the significant improvements in technological breakthroughs for brain imaging and brain-targeted therapies, the treatments of central nervous system (CNS)-associated diseases remain largely ineffective [2]. Although there are new potential therapeutic molecules being continuously discovered, they present a failure of almost 98% for small molecules and 100% for large molecules, being unable to translocate the blood–brain barrier (BBB) [3,4]. The BBB’s unique structure consists of a monolayer of polarized brain endothelial cells interconnected by interendothelial junctions, which comprise adherens junctions and tight junctions [5]. The anatomical and functional features of the BBB are essential for brain homeostasis, maintaining the ionic influx and efflux regulation and protection from harmful substances [6].

Finding new effective approaches to translocate the BBB is challenging. In the last few decades, several efforts have been made to develop new technologies to translocate the BBB, including invasive and non-invasive techniques (e.g., pharmacological and physiological approaches) [7]. The BBB’s overall protective function and anatomical features have to be taken into account in the development of new therapeutic molecules, as delivery systems cannot disrupt and impair its normal function [8,9]. Currently, the most promising approach is the use of nutrient transcytosis mechanisms or other physiological transcytosis pathways or the accumulation at the BBB surface followed by internalization. The design and re-engineering of BBB-crossing peptides, using either receptor-mediated transcytosis (RMT) or adsorptive-mediated transcytosis (AMT), places peptides among the most promising potential drug-delivery systems [10]. However, RMT has disadvantages related to receptor saturation and expression of receptors in different tissues, leading to safety issues.

Cell-penetrating peptides (CPPs) allow a uniform, safe and efficient drug distribution into the brain [7,8,11]. Opposite to the majority of the existing delivery systems, CPPs efficiently transport macromolecules across cellular membranes without toxic and immunogenic effects [12]. These peptides allow rapid delivery of cargo into cells, acting as vectors for small RNA/DNA molecules, plasmids, other peptides, proteins, antibodies and nanoparticles either in vitro or in vivo [13,14]. CPP’s uptake ability depends on a wide range of factors such as their size, biochemical properties, tri-dimensional conformation, and amino acid composition and sequence [12,15].

Since the discovery of the first protein that could enter cells, a cationic protein derived from the human immunodeficiency virus type-1 (HIV-1) protein Tat [16], several functional CPPs with different physicochemical properties and heterogeneous sources were discovered. The vast majority of those peptides have typically five to 30 amino acid residues, are water soluble, amphipathic and nearly all are positively charged at physiological pH. These properties permit the electrostatic interactions between CPPs and the negatively charged glycosaminoglycans (GAGs) and sialic acid residues exposed on the surface of cellular membranes [17,18,19]. Very few anionic peptides have been reported [20,21,22].

The structural diversity and type of cargo carried by CPPs contribute to two main cellular-uptake mechanisms: the energy-dependent endocytosis system and energy-independent direct translocation [23,24]. Endocytosis is a metabolic energy-dependent cellular-uptake mechanism that includes several pathways, such as macropinocytosis, micropinocytosis, clathrin-dependent endocytosis and caveolae/lipid raft-mediated endocytosis [11,25]. This cellular entry mechanism was proposed for arginine-rich peptides, such as HIV-1 Tat-derived peptides and polyarginines [26]. Alternative direct penetration through cellular membranes can occur, which is a so-called energy-independent cellular-uptake mechanism. Direct penetration is highly affected by the cellular membrane heterogeneity, which results in different uptake forms depending on each cell type involved: the carpet and the barrel-stave models, the inverted micelle model and the electroporation-like model [11,12]. For instance, penetratin (a well-studied CPP derived from the Antennapedia homeodomain protein of *Drosophila*) uptake has been associated with direct penetration pathways [27]. Characterization of direct penetration was also thoroughly studied for Pep-1, a chimeric peptide that is composed by a hydrophilic domain that promotes the initial contact with the lipid layer, and a hydrophobic, tryptophan-rich domain, responsible for insertion into the membrane core [28]. Despite the differences between both cellular internalization mechanisms, endocytosis routes and direct penetration, they share similar steps: membrane interaction, membrane permeation and CPP release into the cytoplasm.

It is worth stressing that cell penetration differs from cell translocation, meaning that a CPP can be competent in intracellular delivery but fail in epithelial translocation [29]. For instance, the ten most widely studied membrane-active peptides have a tendency to accumulate unspecifically into different cell lines and, consequently, in different organs, leading to undesired effects [30]. Not all efficient CPPs are BBB-translocators. In addition, standardized protocols for evaluation of the mechanism of uptake and uptake assessment are scarce. For instance, a study from Illien et al. shows the quantification of internalization of three different well-characterized peptides (penetratin, TAT and R9), highlighting the drawbacks of different techniques [31].

Dengue virus type-2 capsid (DEN2C) protein-derived membrane-active peptides have been thoroughly studied by our group. For instance, two distinct CPPs have been found, PepR and PepM, which showed the capacity to target and deliver nucleic acids into cells [32]. An additional study was performed using the four α-helical domains of the DEN2C protein (α1, α2, α3 and α4) to identify trans-BBB peptides. PepH3 α3 sequence (AGILKRW) achieved high brain uptake [29]. PepH3, a hydrophobic peptide, is able to transcytose across the cell, while other peptides accumulate inside the endothelial membrane. It was proposed that PepH3 penetrates the brain using AMT [29].

In this study, we present an anionic trans-BBB peptide the same size as PepH3 (seven amino acid residues), PepNeg sequence (SGTQEEY). PepNeg was designed using PepH3 as a template. All non-polar residues were replaced by polar residues, and basic residues (arginine and lysine) were replaced by glutamic acid residue, rendering the peptide a “negative picture” of PepH3, with an overall negative charge. The PepNeg BBB translocation and internalization mechanism was tested in vitro, using an immortalized mouse brain endothelial cell line bEnd.3, and compared to PepH3. The results show that PepNeg efficiently transports cargo through the BBB model without disrupting the barrier. To date, electrostatic attraction-independent mechanisms for cellular penetration have been described for other negative CPPs [22,33], however, none of these peptides have been tested for BBB translocation. Here, we describe the first anionic trans-BBB peptide, which implies a rethinking of the role of electrostatic attraction in BBB translocation.

## 2. Results

**Figure 1:**
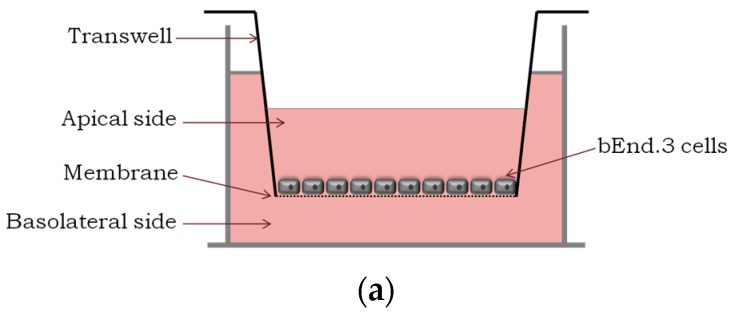

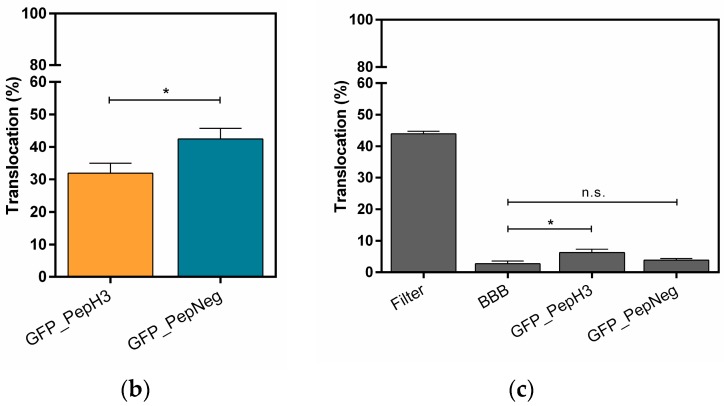
Translocation across a BBB model and the integrity study. (**a**) An in vitro BBB model consisting of an immortalized mouse brain endothelial cell line bEnd.3 grown as a monolayer on the apical side of the tissue culture insert; (**b**) Percentage of translocation determined by GFP fluorescence intensity measurements, for 0.1 μM of GFP_PepH3 (orange) and GFP_PepNeg (blue). A one-way ANOVA statistical test followed by a Dunnett’s test was used to compare both fluorescence measurements obtained (* *p* ≤ 0.05); (**c**) Percentage of FD40 permeability after exposure to GFP_peptide (for 5 h). The recorded percentages were compared to two controls: one with the transwell without cells (“Filter”) and the other consisting of the BBB model without GFP_peptide incubation (“BBB”). The statistical test used to compare each fluorescence measurement with the BBB fluorescence obtained was a one-way ANOVA followed by a Dunnett’s test (* *p* ≤ 0.05; n.s., not significant). The values were obtained from duplicates of three independent experiments.

PepNeg trans-BBB potential to deliver a cargo (green fluorescent protein (GFP)) was evaluated in an in vitro BBB transwell model using GFP fluorescence emission intensity measurements (Figure 1a). Both peptides (PepH3 and PepNeg) were recombinantly produced in *E. coli* as an N-terminal fusion with GFP (27 kDa). In addition, a spacer of glycine and serine (SGGGGSGGGGSS) was added to give flexibility to the peptide in the final construct. The specific GFP_PepNeg cellular translocation pathway was screened by selective inhibition of endocytosis through temperature block and specific organelle inhibitors. The results obtained for PepNeg were compared to PepH3 using the same conditions.

### 2.1. Translocation Across an In Vitro Model of BBB

An in vitro BBB model consists of brain endothelial cells growing on the apical side of a porous membrane positioned between two compartments (Figure 1a). Herein, an immortalized mouse brain endothelial cell line bEnd.3, well-established as a model for the BBB [34,35,36], was used to evaluate peptide translocation.

0.1 μM of GFP_peptide (PepH3 or PepNeg) was added to the apical side of the BBB model and total volume in the basolateral side collected after 5 h of incubation. The GFP_peptide translocation was quantified by measuring the fluorescence intensity in the basolateral side. In addition, the permeability of the BBB model was measured using fluorescein isothiocyanate-conjugated dextran (FD40, with molecular weight of 40 kDa) [37]; low or no permeability of FD40 excludes the occurrence of paracellular transport.

The results show that after 5 h, 31.91 ± 3.02% of GFP_PepH3 translocates the BBB, being in the basolateral chamber (Figure 1b; orange). The translocation of GFP_PepNeg was 1.3-times higher (42.50 ± 3.28%) than for GFP_PepH3 (Figure 1b; blue). Independently of the peptide global net charge, both peptides are able to cross the endothelial barrier carring a hydrophobic and negatively charged fluorophore, the GFP [38]. Furthermore, evaluation of BBB integrity revealed that GFP_PepH3 had minimal influence on the barrier permeability, and consequently, minimal effects on tight junction disruption, avoiding paracellular leakage. Cells incubated with GFP_PepNeg had even lower permeability to FD40, with percentage of translocation similar to control cells without peptides (“BBB”, Figure 1c), thus safer (Figure 1c).

### 2.2. In Vitro Determination of Intracellular Mechanism of Peptide Translocation

**Figure 2:**
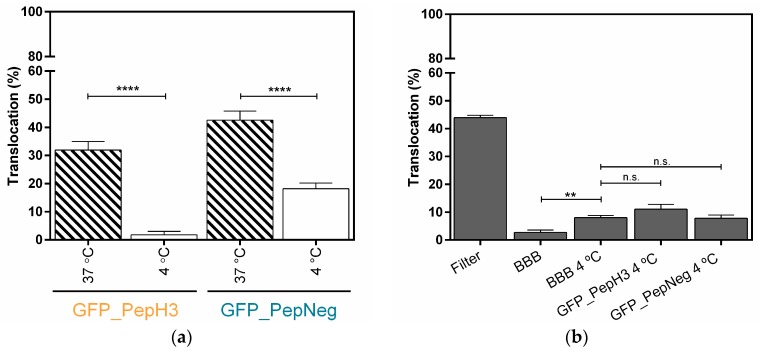
Metabolic inhibition studies of GFP_PepH3 and GFP_PepNeg cellular-translocation pathways and the model-integrity study. (**a**) Percentage of translocation for GFP_PepH3 (orange) and GFP_PepNeg (blue), determined by GFP fluorescence intensity detection, at 37 °C (dashed) and at 4 °C (white). A one-way ANOVA statistical test followed by a Dunnett’s test was used to compare each fluorescence measurement at 4 °C with the respective experiments at 37 °C (**** *p* < 0.0001); (**b**) Percentage of FD40 translocation determined by fluorescence intensity detection. The recorded percentages were compared to three controls: one with the transwell without cells (“Filter”) and the others consisting of the BBB model with no peptide incubation at 37 °C (“BBB”) and at 4 °C (“BBB 4 °C”). The statistical test used to compare the fluorescence measurements obtained was a one-way ANOVA followed by a Dunnett’s test (** *p* ≤ 0.01; n.s., not significant). The values were obtained from duplicates of three independent experiments.

A systematic analysis of the peptide-translocation mechanism across an in vitro BBB model was achieved by inhibition of one or more transport pathways. Firstly, a temperature block was tested, as energy-dependent mechanisms are nearly inhibited at 4 °C. Therefore, the transcellular transport at 4 °C and 37 °C (control) of GFP_PepH3 and GFP_PepNeg were determined by fluorescence intensity measurements (Figure 2a). For GFP_PepH3, translocation across the BBB model decreased from 31.91 ± 3.02% at 37 °C to 1.82 ± 1.20% at 4 °C (Figure 2a, orange). PepNeg was able to transport GFP throught the BBB model at both temperatures, although the efficiency at 4 °C was much lower than at 37 °C (18.17 ± 2.00% and 42.50 ± 3.28%, respectively; Figure 2a, blue).

The metabolic inhibition studies demonstrated that GFP_PepH3 translocation is inhibited at low temperatures, suggesting that the GFP_PepH3 translocation mechanism is energy-dependent. On the other hand, at 4 °C there is a significant impairment but no inhibition of GFP_PepNeg translocation in comparison with the experiment preformed at 37 °C, indicating that this peptide crosses the BBB model using both energy-dependent and -independent mechanisms. The influence of temperature on the BBB integrity was further evaluated. As shown in Figure 2b, incubation at 4 °C resulted in a slight increase in permeability compared to the values at 37 °C. However, no significant changes were observed when the peptides were added to the BBB at 4 °C.

To further understand the details of the mechanism of translocation of GFP_PepH3 and GFP_PepNeg, the effect of endocytic inhibitiors was evaluated. Therefore, methyl-β-cyclodextrin (MβCD) was used to inhibit the cholesterol-dependent endocytic patways interfering with the lipid raft/caveolin-mediated endocytosis [39]; dynasore was employed to inhibit dynamin, an essential protein for vesicle scission for both clathrin- and caveolin-dependent endocytosis [40]; chlorpromazine was used to restrict clathrin-dependent endocytosis [40]; 5-(*N*-ethyl-*N*-isopropyl) amiloride (EIPA) was used to block macropinocytosis and brefeldin A [41], a protein that impairs Golgi trafficking, was employed to inhibit vesicle formation and hinder peptide translocation. 

**Figure 3:**
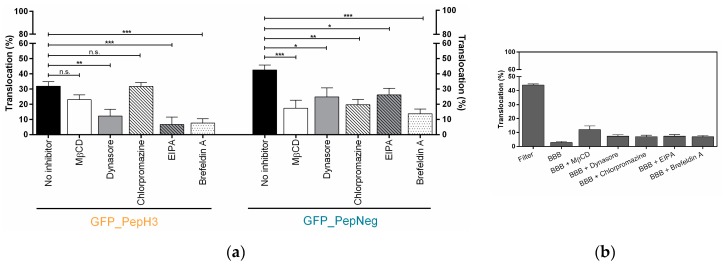
Screening of GFP_PepH3 and GFP_PepNeg translocation, in the presence of endocytosis inhibitors, and the barrier-integrity studies. (**a**) Percentage of translocation for GFP_PepH3 (orange) and GFP_PepNeg (blue), determined by GFP fluorescence intensity detection at 37 °C in the presence of the following inhibitors: MβCD (5 mM, white bars), dynasore (50 µM, grey bars), chlorpromazine (50 µM, white-dashed bars), EIPA (100 µM, grey-dashed bars) and brefeldin A (10 µg/mL, white-dotted bars). A one-way ANOVA statistical test followed by a Dunnett’s test was used to compare each fluorescence measurement with the respective experiments at 37 °C with no-inhibitor (black bars; * *p* ≤ 0.05; ** *p* ≤ 0.01; *** *p* ≤ 0.001; n.s., not significant); (**b**) Percentage of BBB permeability to FD40, determined by fluorescence intensity measurements. The recorded percentages were compared to two controls: the transwell without cells (“Filter”) and the other consisting of the barrier model without peptide at 37 °C (“BBB”). The values were obtained from duplicates of two independent experiments.

For GFP_PepH3, the translocation was significantly inhibited with dynasore (Figure 3a, orange), decreasing from 31.91 ± 3.02% to 12.34 ± 4.36%, which means that one or both dynamin-related pathways, clathrin- and caveolin-mediated endocytosis, play an important role in peptide translocation. Additionally, those routes were tested individually. The incubation with chlorpromazine and MβCD resulted in a slight decrease in peptide translocation, but inhibition was not statistically significant (from 31.91 ± 3.02% to 31.79 ± 2.60% and to 23.05 ± 3.12%, respectively). Incubation with MβCD resulted in an increase in permeability to FD40 (Figure 3b), indicating an increase in paracellular leakage. Furthermore, the GFP_PepH3 translocation mechanism was also significantly inhibited by EIPA (6.79 ± 4.83%) and brefeldin A (7.71 ± 2.78%). Apart from MβCD, the inhibitors tested had no significant effect on the BBB permeability to FD40 (Figure 3b).

GFP_PepNeg translocation through the BBB in the in vitro model was impaired by the different inhibitory conditions tested. Incubation with dynasore resulted in lower BBB translocation (24.83 ± 5.86%), when compared to no-inhibitor (42.50 ± 3.28%), suggesting that one or both of the dyamine-dependent routes may be involved. In addition, the translocation of GFP_PepNeg in the presence of chlorpromazine (19.67 ± 3.52%) and MβCD (17.41 ± 5.22%) was also inhibited. A less-pronouced effect was observed with EIPA, with a decrease of GFP_PepNeg translocation of less than 16% (26.09 ± 4.41%). A significant decrease in GFP_PepNeg translocation was also obtained in the presence of brefeldin A (13.75 ± 3.03%) (Figure 3a).

## 3. Discussion

CPPs play an important role in the development of new therapeutic agents, since they are able to carry therapeutic molecules across cell membranes and occasionaly through an endothelium, such as the BBB, with no significant cellular damage [42]. Here, we have discovered the first anionic peptide that has the capacity to deliver a cargo across an in vitro BBB model. GFP_PepNeg presents higher trans-BBB efficacy than GFP_PepH3. The PepH3 ability to cross the BBB was demonstrated both in vitro and in vivo [29]. Transport of GFP by both PepH3 and PepNeg is transcellular, since integrity of the BBB is mantained. Transcellular transport is the primary mechanism by which most molecules cross the normally restrictive BBB. This transcellular transport might occur through transcytosis or diffusion, only observed for lipophilic molecules. Transcytosis is the result of endocytosis (visicle budding and fission) at the apical membrane and exocytosis (membrane fusion and release of vesicular contents) at the basolateral membrane [43]. It has been proposed that cellular uptake of CPPs is receptor independent [44]. The interaction of CPPs with cells might be initiated by binding to glycoproteins containing negatively-charged moieties such as GAGs [45,46]. GAGs are ubiquitously expressed on cell surfaces and are involved in cell signaling, regulation and binding [46,47]. Negatively charged groups present on GAGs include carboxylates and sulfates. Heparan sulfate (HS) GAGs have been indentified as important moieties for cationic-peptide uptake [48]. The translocation through diffusion is relatively fast and this mechanism can be confirmed by performing experiments at 4 °C, where endocytosis is inhibited, or through endocytosis inhibitors. Herein, we report a systematic study of GFP_PepH3 and GFP_PepNeg translocation of the BBB, using a temperature block (4 °C) and treatment of cells with endocytosis-specific inhibitors.

GFP_PepH3 moves through the intact BBB via AMT, mediated by macropinocytosis and caveolin-mediated endocytosis, confirmed by inhibition with EIPA and MβCD. In addition, the transport through the cell is dependent on vesicule formation to and from the Golgi apparatus or endoplasmic reticulum (inhibition with brefeldin A), followed by exocytosis.

GFP_PepNeg translocation through the BBB involves more than one mechanism. Firstly, transport occurs at 4 °C, suggesting that GFP_PepNeg is able to diffuse through the endothelial barrier. Transport of PepNeg at 4 °C is less efficient (18.17 ± 2.00%), due to reduced cell-membrane fluidity and dynamics [31]. Secondly, the treatment of cells with endocytosis-specific inhibitors reduces the uptake of GFP_PepNeg. Therefore, GFP_PepNeg uses a dynamin-dependent endocytic pathway, including both clathrin- and caveolin-dependent endocytosis. Furthermore, inhibition of vesicle formation influences peptide translocation, revealing an internalization mechanism dependent on the Golgi apparatus or endoplasmic reticulum. Macropinocytosis, a clathrin- and caveolin-independent endocytosis, is also involved in GFP_PepNeg translocation. The first step of interaction of anionic GFP_PepNeg with cell membranes is triggered by an unknown mechanism, possibly through binding to a cationic site at the plasma membrane. GFP_PepNeg might bind to these sites in the form of clusters, probably because of their repulsive interactions with the negatively charged domains of the cell surface. These protein aggregates may induce vesicle budding and fission through the different mechanisms described: macropinocytosis, and clathrin- and caveolin-independent. A recent study from Martín et al. also describes the uptake of a negatively charged peptide (SAP(E)), proposing that internalization occurs through membrane interaction and endocytosis [22]. They suggest that interaction with negatively charged membranes is not a requirement for internalization of SAP(E) [22]. The capacity to use either AMT and/or direct penetration by GFP_PepNeg might be related to peptide concentration at the surface of the cell membrane. For low concentrations of GFP_PepNeg, direct penetration occurs, while the accumulation of peptide at the membrane surface induces internalization via macropinocytosis or caveolin/clathrin endocytosis. However, a description of the mechanisms that rule the interaction with phospholipids at the cell membrane is still missing.

In this work, we present a new anionic peptide extremely efficient for BBB crossing, as well as in delivery cargoes (e.g., GFP), with negligible effects on barrier integrity. Therefore, as PepNeg translocates the BBB using both AMT mechanisms, energy-dependent and -independent mechanisms, the interaction between the peptide and the negatively charged surface of the membrane should not be a requirement for this peptide translocation. This further confirms that CPPs do not need to be positively charged to shuttle molecules through the BBB.

## 4. Materials and Methods

### 4.1. Chemicals and Materials

GFP DNA template was purchased from Nzytech (Lisbon, Portugal), with the following sequence: MGVSKGEELFTGVVPILVELDGDVNGHKFSVSGEGEGDATYGKLTLKFICTTGKLPVPWPTLVTTLTYGVQCFARYPDHMKQHDFFKSAMPEGYVQERTIFFKDDGNYKTRAEVKFEGDTLVNRIELKGIDFKEDGNILGHKLEYNYNSHKVYITADKQKNGIKVNFKTRHNIEDGSVQLADHYQQNTPIGDGPVLLPDNHYLSTQSALSKDPNEKRDHMVLLEFVTAAGITLGMDELYK.

Specific primers, NheI and XhoI restriction enzymes and T4 DNA ligase were purchased from Thermo Fisher Scientific (Waltham, MA, USA). Tris-borate-EDTA (TBE) buffer, agarose ultrapure grade, Tris-acetate-EDTA (TAE) buffer, green safe DNA intercalator and *Escherichia Coli* NZY5α and BL21 (DE3) competent cells were purchased from Nzytech (Lisbon, Portugal). pET-28a-c (+) vector (69864-3), KOD hot start master mix and benzonase nuclease were purchased from Novagen (Merck, Darmstadt, Germany). Kanamycin sulfate antibiotic, imidazole and fibronectin were purchased from Calbiochem (Merck, Darmstadt, Germany). Isopropyl-beta-thiogalactopyranoside (IPTG), 1,4-dithiothreitol (DTT), bovine serum albumin (BSA) protein and the fluorescein isothiocyanate-dextran with molecular weight of 40 KDa (FD40) were purchased from Sigma-Aldrich (Merck, Darmstadt, Germany). Glycerol anhydrous was obtained from AppliChem (Darmstadt, Germany). Sodium chloride and sodium phosphate were purchased from EMSURE (Merck, Darmstadt, Germany). Complete EDTA-free protease inhibition cocktail was obtained from Roche (Basel, Switzerland).

The bEnd.3 brain endothelial cell line was obtained from American Type Culture Collection (Manassas, VA, USA). Dulbecco’s Modified Eagle’s Medium (DMEM) with (41966-029) or without phenol red (21063029), fetal bovine serum (FBS) and penicillin–streptomycin were purchased from Gibco (Thermo Fisher Scientific, Waltham, MA, USA). Fibronectin bovine plasma was obtained from Calbiochem (Merck, Darmstadt, Germany). Trypsin-EDTA was purchased from Sigma-Aldrich (Merck, Darmstadt, Germany). All inhibitory compounds were purchased from Sigma-Aldrich (Merck, Darmstadt, Germany).

### 4.2. Peptide Conjugates Preparation

The recombinant proteins GFP_PepH3 and GFP_PepNeg were amplified by PCR with the primers: PepH3 (3′ CTC CAT ATG GCT AGC ATG GGA GTT AGC AAA GGT GAA G 5′ and 5′ CCG CAC CTC GAG CTA AGA CCA ACG TTT CAG AAT GCC CGC ACT GCT GCC ACC GCC ACC GGA GCC ACC GCC ACC ACT TTT GTA CAG TTC ATC CAT GCC 3′) and PepNeg (3′ CTC CAT ATG GCT AGC ATG GGA GTT AGC AAA GGT GAA G 5′ and 5′ CCG CAC CTC GAG CTA AGA GTA TTC TTC CTG GGT GCC GGA ACT GCT GCC ACC GCC ACC GGA GCC ACC GCC ACC ACT TTT GTA CAG TTC ATC CAT GCC 3′), adding 5′ NheI and 3′ XhoI restriction sites. The resulting PCR products were gel-purified, digested with NheI/XhoI restriction enzymes and cloned into pET-28a-c (+) vector. The recombinant pET-28a-c (+) vector was transformed into *Escherichia coli* NZY5α competent cells by the heat-shock method. After transformation, positive colonies were tested by colony-PCR and the insertion of the recombinant sequence verified by Sanger DNA sequencing (GATC Biotech, Constance, Germany). The PCR products were confirmed on an agarose gel. Finally, *Escherichia coli* BL21 (DE3) competent cells were used to transform the correct recombinant pET28 vector constructed using the heat-shock method.

### 4.3. Recombinant Fusion Protein Expression and Purification

For expression of the recombinant fusion proteins, the clones selected were grown overnight in super broth (SB) medium (30 mg/mL of tryptone, 20 mg/mL of yeast extract and 10 mg/mL of 3-(*N*-Morpholino)propanesulphonic acid (MOPS)) supplemented with kanamycin (50 μg/mL), in a shaker incubator (VWR^®^ shaker incubating 230 V, product code: 12620-948, Philadelphia, PA, USA) at 37 °C and 220 rpm. Then, cultures were diluted ~1:25 in fresh SB medium containing kanamycin sulfate antibiotic (50 μg/mL), incubated at 37 °C and 220 rpm until the OD_600_ reached 0.6. Protein expression was induced with 0.6 mM of IPTG and growth was performed for 6 h at 37 °C and 220 rpm. Cells were harvested by centrifugation in Beckman J2-21M/E High Speed Centrifuge (Indianapolis, IN, USA) rotor JA-14 (4000 rpm for 15 min at 4 °C), resuspended in 25 mL of binding buffer and frozen at –20 °C until protein purification was performed.

For purification of fusion proteins, benzonase (1 U/mL) was added to protein extract and incubated on ice for 20 min. After cell lysis by sonication, the protein extract was recovered by centrifugation in Eppendorf Centrifuge 5418 R (Hamburg, Germany) rotor FA-45-18-11 (13,000 rpm for 45 min at 4 °C). Protein purification was performed using nickel-chelating affinity chromatography via the Histrap HP column (GE Healthcare Life Sciences, Chicago, IL, USA) connected to an AKTA^®^ Explorer chromatographic system (GE Healthcare Life Sciences, Chicago, IL, USA). Cell-free extracts were loaded in the affinity column previously equilibrated with column buffer (50 mM sodium phosphate, 1 M NaCl, 10% glycerol, pH = 7.6). Column was then washed with 60 mM imidazole and the recombinant fusion proteins eluted through a linear imidazole gradient using a 500 mM imidazole buffer (50 mM sodium phosphate, 1 M NaCl, 500 mM imidazole and 10% glycerol). A final purification step was performed using size-exclusion chromatography in a Sephadex^®^ G200 column (GE Healthcare Life Sciences, Chicago, IL, USA) equilibrated with phosphate-buffered saline (PBS). The purified proteins were stored frozen at −80 °C in PBS buffer, supplemented with 1 mM of DTT and 5% of anhydrous glycerol.

Protein concentration was determined by the Bradford method as described elsewhere [49], using bovine serum albumin as reference standard.

### 4.4. Cell Culture

bEnd.3 brain endothelial cells (ATCC, CRL-2299) were cultured in DMEM supplemented with 10% FBS and 1% penicillin-streptomycin in a humidified atmosphere of 5% CO_2_ and 95% air at 37 °C. Cells were adhered in monolayers and, when confluent, were harvested using trypsin-EDTA and 3500 cells/well were then seeded in PET membrane filters (1 μm pore size) and pre-coated with fibronectin bovine plasma (3 μg/mL) for 24-well cell culture plates (BD Falcon). Cells were allowed to reach a confluent monolayer for 10 days and medium changed every two days, which allowed the formation of stable tight junctions mimicking the natural BBB [50].

### 4.5. In Vitro Translocation and Integrity Studies

Fusion proteins were previously diluted in DMEM without phenol red to a final concentration of 0.1 μM and added to the apical side of bEnd.3 cells grown in tissue-culture inserts and incubated for 5 h. After incubation, recombinant proteins were collected from the basolateral side of the cellular membrane and fluorescence was measured at 395 nm excitation and 509 nm emission wavelengths using the Infinite F200 TECAN plate reader.

The percentage (%) of translocation was calculated using the following Equation (1):

Translocation (%) = Fi/Fp × 100.(1)

Fi stands for the fluorescence intensity recorded from the basolateral side of the tissue-culture insert and Fp for the fluorescence intensity of total protein added to the apical side.

Then, after 5 h incubation with the peptide solution, in vitro integrity studies were performed. Herein, FD40 was diluted in DMEM without phenol red from a stock solution of 25 mg/mL, to a final absorbance below 0.1. The fluorescent probe was added to the apical side and incubated for 2 h. After incubation, samples were collected and fluorescence measured at 493 nm excitation and 560 nm emission wavelengths using the Infinite F200 TECAN plate reader. The percentage of translocation was determined using Equation (1).

### 4.6. Metabolic and Endocytosis Inhibition Studies in an In Vitro Model of BBB

To determine endocytic pathway, pharmacological inhibitors of the three major routes of endocytosis were identified: clathrin-dependent and -independent endocytosis were induced by adding 50 µM of chlorpromazine and 50 µM of dynasore, respectively; EIPA (100 µM) to inhibit macropinocytosis; and 5 mM of MβCD to block the lipid raft/caveolae-mediated endocytosis. In addition, breferdin A (10 µg/mL) was also used to interfere with Golgi vesicle formation. Cells were exposed to different concentrations of the inhibitors for 30 min prior to incubation with 0.1 μM of either GFP_PepH3 or GFP_PepNeg. After peptide incubation, the fluorescence signal of all fractions was measured with Infinite F200 plate reader (TECAN, Grodig, Austria) using GFP excitation and emission wavelengths.

The integrity of the BBB was evaluated for all inhibitory conditions using a FD40 probe, as previously described.

Metabolic inhibition studies were also performed. To assess the effect of the temperature, cells were incubated with 0.1 μM of either GFP_PepH3 or GFP_PepNeg for 5 h at 4 °C.

## 5. Conclusions

The in vitro results showed that the anionic peptide, PepNeg, has high ability to transport cargoes through a BBB model without disrupting the BBB, presenting higher translocation than that reported for *trans*-BBB peptide PepH3. The mechanisms of translocation are different for each peptide; for instance, GFP_PepH3 enters cells using energy-dependent mechanisms, while GFP_PepNeg uses both energy-dependent and -independent pathways. Thus, PepNeg is capable of diffusing through the BBB carrying a large cargo, GFP (27 kDa). Therefore, a new anionic trans-BBB peptide is presented with unique properties. We foresee several trans-BBB applications for PepNeg, in particular in the therapy of central nervous system-related diseases.
